# Biological Activity of Genus Hypericum Sect. *Hypericum* Species—*H. tetrapterum*, *H. maculatum* subsp. *immaculatum*, *H. triquetrifolium*

**DOI:** 10.3390/molecules28176218

**Published:** 2023-08-24

**Authors:** Nebojša Kladar, Biljana Božin, Katarina Bijelić, Mirjana Bogavac, Maja Karaman, Branislava Srđenović Čonić, Milica Rat, Goran Anačkov

**Affiliations:** 1 Department of Pharmacy, Faculty of Medicine, University of Novi Sad, Hajduk Veljkova 3, 21000 Novi Sad, Serbia; 2 Center for Medical and Pharmaceutical Investigations and Quality Control, Faculty of Medicine, University of Novi Sad, Hajduk Veljkova 3, 21000 Novi Sad, Serbia; 3 Clinical Center of Vojvodina, Department of Obstetrics and Gynecology, Faculty of Medicine, University of Novi Sad, Hajduk Veljkova 3, 21000 Novi Sad, Serbia; 4 Department of Biology and Ecology, Faculty of Sciences, University of Novi Sad, Trg Dositeja Obradovica 2, 21000 Novi Sad, Serbia

**Keywords:** *Hypericum*, antihyperglycemic, monoaminoxidases, acetylcholinesterase, antimicrobial, HPLC-DAD

## Abstract

St. John’s wort (*Hypericum perforatum*, Hypericaceae) has long been used in traditional medicine as a potent remedy, while many other species of this genus have not been thoroughly investigated. The study aimed to detect the biological activity, including antioxidant, antihyperglycemic, anticholinergic, antimicrobial and monoaminoxidase inhibitory potential, of water-alcoholic extracts of three species autochthonous for Serbia and Greece from plant genus *Hypericum* (section *Hypericum*—*H. tetrapterum*, *H. maculatum* ssp. *immaculatum* and *H. triquetrifolium*), followed by phytochemical profiling. The highest amount of phenolics was recorded in *H. maculatum* subsp. *immaculatum* extract, while the highest abundance of flavonoids was characteristic of *H. tetrapterum* extract. Hypericin and hyperforin, quercetin, and its flavonoid, rutin, were present in all of the evaluated species. The evaluated species were good scavengers of DPPH, OH and NO radicals, as well as potent reducers of ferric ions in FRAP assay. Furthermore, the evaluated species were shown as potent inhibitors of monoaminoxidase A and α-glucosidase and modest inhibitors of acetylcholinesterase, monoaminoxidase B and α-amylase. No anti-Candida activity was recorded, but the extracts were effective against MRSA *Staphylococcus aureus* and *Enterococcus* sp., as well as against *Proteus mirabilis*. The obtained results strongly highlight the need for further in vivo studies in order to better define the potential of the medicinal application of the studied species.

## 1. Introduction

The genus *Hypericum* includes more than 500 species, widely geographically distributed, classified into 36 taxonomic sections [1]. The best-studied representative of the genus is St. John’s wort (*H. perforatum*, Hypericaceae), known for its long history of traditional application as a potent remedy. Basically, preparations based on St. John’s wort are being used in two forms, water-ethanolic extracts and oil macerates made of upper areal parts of *H. perforatum*. The first one has been shown to be clinically effective in the treatment of mild to moderate forms of depression, while oil macerate is being used traditionally externally for the treatment of wounds, bruises and eczema, as well as internally for the treatment of gastric ulcers [2,3]. Several classes of compounds with promising biological potential are present in *H. perforatum*. The most specific are naphthodianthrones (hypericin, pseudohypericin) and phloroglucinols (hyperforin, adhyperforin). Furthermore, some more common classes of secondary metabolites are present, such as phenolic acids (gallic, chlorogenic, caffeic and ferulic acid), flavonoids and their glycosides (quercetin, rutin, hyperoside), biflavonoids (amentoflavone) and xanthones [2]. These compounds are responsible for various biological activities of *H. perforatum*, such as antioxidant, antimicrobial, anticholinesterase, antihyperglycemic and photodynamic activities. However, phytochemical studies of the genus *Hypericum* have shown that the presence of the previously mentioned secondary metabolites is not *species*-specific and indicated the resemblance of the qualitative and quantitative chemical profile of other *Hypericum* species with the official biological source of the herbal drug (*Hyperici herba*) [4,5]. Furthermore, some of the previously mentioned compounds can be found in quantities several times higher in various representatives of the genus *Hypericum* other than *H. perforatum*. This emphasizes the importance of further studies on the biological potential of these species [2,6]. On the other hand, it is not uncommon that *Hyperici herba* samples often contain *Hypericum* species other than the official biological source, which is a direct consequence of herbal material collectors’ inability to botanically recognize *H. perforatum*. The taxa analyzed in this paper are representatives of a typical section of the genus that are characterized by the moderate growth of erect stems and belong to the life-form hemicryptophytes. *Hypericum tetrapterum* is distributed in the Eurasian region, in mountain and subalpine ranges, in humid habitats, while the *H. maculatum* subsp. *immaculatum* can be found in the same altitude range but with narrower distribution limited to the mountain massifs of the Balkan Peninsula and southern Carpathians [7]. On the other hand, *Hypericum triquetrifolium* (curled-leaved St. John’s-wort) is an eumediterranean species inhabiting Mediterranean countries and spreading to western Iran [8].

Therefore, the aim of the current study was to chemically characterize three representatives of the genus *Hypericum*, section *Hypericum* (*H. tetrapterum*, *H. maculatum* ssp. *immaculatum* originated from Serbia and *H. triquetrifolium* originated from Greece), as well as to evaluate their biological potential in terms of antioxidant, antihyperglycemic, anticholinergic, antimicrobial and monoaminoxidase inhibitory potential.

## 2. Results and Discussion

### 2.1. Chemical Characterization of Hypericum Extracts

The results of the preliminary chemical characterization (Table 1) show that the amount of total phenolic and total flavonoids was in the ranges 83.52–194.24 mg GAE/g dried extract (d.e.) and 24.76–58.15 mg QE/g d.e., respectively. The recorded values correspond to previous analyses of these species [9,10], as well as to analyses of *H. perforatum* extracts [11,12]. The highest amount of phenolics was recorded in *H. maculatum* subsp. *immaculatum* extract, while the highest abundance of flavonoids was characteristic of *H. tetrapterum* extract. Detailed chemical profiling of the obtained extracts indicated the presence of several classes of compounds (Table 1). Hypericin and hyperforin were present in all of the collected plants, while the lowest recorded amounts were characteristic of *H. maculatum* ssp. *immaculatum*. To the best of our knowledge, this is the first report of phytochemical screening of *H. maculatum* ssp. *immaculatum*, while for the other two evaluated species, the amounts of the previously mentioned compounds generally correspond to published studies [9,13]. Rutin, and its flavonoid quercetin, were also detected in all of the analyzed samples, and the obtained results correspond to the previously published studies [14,15]. However, the pattern of accumulation of the mentioned compounds is interesting. It seems that *H. tetrapterum* and *H. triquetrifolium* predominantly accumulate in the glycoside form (H(2, 15) = 12.5 *p* = 0.002), while the aglycone is more abundant in *H. maculatum* ssp. *immaculatum* (H(2, 15) = 12.02 *p* = 0.002). Further, biflavonoid, amentoflavone, was only not detected in *H. maculatum* ssp. *immaculatum*, which could represent a chemotaxonomic marker for differentiation from ssp. *maculatum* [5]. Furthermore, caffeic, *p*-hydroxybenzoic, ferulic and chlorogenic acid, were the most abundant in *H. maculatum* ssp. *immaculatum*. Taking into account the results obtained for the three evaluated *Hypericum* species and the non-questionable influence of abiotic factors on the intensity of secondary metabolites synthesis in plants, it can be concluded that there is a high level of chemical profile resemblance of *H. tetrapterum* and *H. triquetrifolium* with the official source of the herbal drug (*H. perforatum*). On the other hand, all three evaluated species are characterized by a higher abundance of quercetin and rutin [12,13,15].

**Figure 1:**
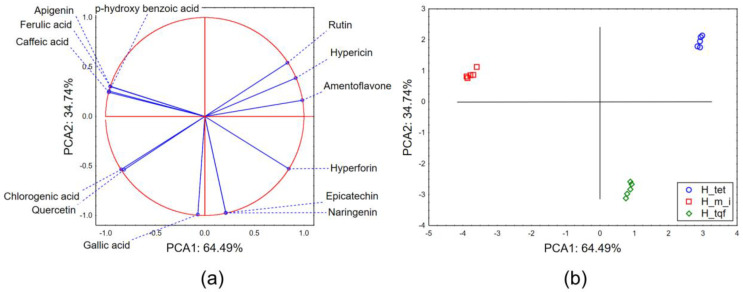
PCA—secondary metabolites: (**a**) PCA loadings, (**b**) the position of the evaluated extracts in the space defined by the first two principal components (PCAs).

The results of principal component analysis applied to the dataset describing the quantities of evaluated secondary metabolites in the analyzed extracts show that the first two principal components (PCAs) describe more than 99% of the sample’s variability. In terms of the first principal component (PCA1), most of the variability is described by the quantified amounts of hypericin, hyperforin, rutin, amentoflavone and phenolic acids (ferulic, caffeic and *p*-hydroxybenzoic acid) (Figure 1a). The shape of the recorded variability in terms of the second principal component (PCA2) mostly correlates with the quantified amount of gallic acid. The position of the evaluated extracts in the space defined by the first two principal components (Figure 1b) shows a grouping of *H. tetrapterum* (H_tet) and *H. triquetrifolium* samples (H_tqf) in the positive part of PCA1 as a result of the higher recorded amount of hypericin, hyperforin, rutin and amentoflavone. The separative grouping of H_tet and H_tqf samples in terms of PCA2 is a result of higher amounts of epicatechin and naringenin in H_tqf samples and the dominance of hypericin, rutin and amentoflavone in H_tet samples. *H. maculatum* ssp. *immaculatum* (H_m_i) samples are located in the negative part of PCA 1 as a result of the moderate quantified amount of rutin, hypericin and hyperforin, but the higher abundance of caffeic, *p*-hydroxybenzoic and ferulic acids.

### 2.2. Biological Potential of Evaluated Hypericum Species

#### 2.2.1. Antioxidant Potential

The studies conducted in the last decades have identified plants and plant preparations as valuable sources of antioxidants. This has induced their application in the prevention and treatment of various pathological conditions, as well as utilization predominantly in the food and cosmetics industries. However, the complexity of the oxidative processes and the nature of generated free radicals demands the application of several antioxidant assays in order to comprehensively assess the antioxidant properties of specific agents [11,12]. In our study, we have applied five antioxidant assays with the aim of critically evaluating the potential of the examined *Hypericum* species extracts to scavenge free radicals and inhibit the oxidative processes. The obtained results (Table 2) were also compared with the antioxidant potential of substances already recognized for their antioxidant potential, evaluated under the same experimental conditions.

The evaluated *Hypericum* extracts have exhibited a strong scavenging potential of DPPH radical, with RSC_50_ values in the range 1.93–3.54 µg/mL, which was also demonstrated in the previously conducted studies [16,17,18,19] and is further confirmed by the IC_50_ values obtained for quercetin dihydrate and propyl gallate (Table 2). Although there were reports stating that hypericins could be responsible for the antioxidant effect of *Hypericum* species [20], it is much more likely that this activity is a result of phenolic acids and flavonoid presence [19]. Namely, highly abundant flavonoids and flavonoid glycosides in *Hypericum* species are quercetin and its derivatives, which are known for their excellent radical scavenging capacity [19]. A somewhat weaker antioxidant potential was recorded in the case of the evaluation of the neutralization of nitroso and hydroxyl radicals, where the calculated RSC_50_ values ranged from 12.11 to 32.17 µg/mL and from 51.74 to 58.74 µg/mL, respectively, which correspond to previous studies [17]. This claim is further supported by the results of the antioxidant potential obtained for propyl gallate, butylated hydroxytoluene and ascorbic acid under the same experimental conditions (Table 2). However, it must be taken into account that the comparison is being made between antioxidants, which are pure compounds, and herbal extracts, which are complex mixtures of a large number of compounds, of which some do not possess antioxidant activity. Furthermore, the evaluation of the ability of the examined extracts to reduce Fe^3+^ ions has demonstrated strong antioxidant potential, ranging from 113.76 to 176.75 mg of AAE/g d. e. It is worth noticing that the evaluated extracts showed modest potential to inhibit the lipid peroxidation process since the IC_50_ value could be determined for only *H. maculatum* ssp. *immaculatum* (IC_50_ = 514.96 µg/mL) and the other two investigated extracts did not manage to inhibit 50% of this oxidative process. One of the reasons for this could be the polarity of the secondary metabolites present in *Hypericum* extracts and their modest penetration into the lipid membranes. Namely, the previously conducted studies have marked flavonoid aglycones, especially quercetin, as responsible for the inhibition of the lipid peroxidation process [19] regarding its lipophilic nature. It can be noticed that *H. maculatum* ssp. *immaculatum* extract contained the highest amount of quercetin, as well as the highest amounts of phenolic acids (ferulic, gallic, chlorogenic, caffeic and *p*-hydroxybenzoic acids). It is known that cinnamic acid derivatives show stronger antioxidant potential when compared to benzoic acid derivatives [21,22]. Further, it was shown that the specific position of hydroxyl and methoxy groups in caffeic and ferulic acids, respectively, improves their antioxidant potential [21], while gallic and chlorogenic acids have been previously confirmed as strong antioxidants [19,23]. On the other hand, although all of the extracts contained rutin and quercetin, previous studies suggest a significantly higher contribution of quercetin to the antioxidant properties of herbal extracts [24].

#### 2.2.2. Inhibition of Biologically Important Enzymes

##### Inhibition of Acetylcholinesterase, Monoamine Oxidases A and B

The activity of acetylcholinesterase (AChE) and monoaminoxidases A and B (MAO-A and MAO-B) is vital for the regulation of various physiological processes in human organisms. A number of drugs have been developed with the aim of modifying the activity of these enzymes that are vital in the pathophysiology of some of the frequent diseases. The obtained results have demonstrated moderate anticholinesterase and anti-MAO-B activity of the examined *Hypericum* extracts, with IC_50_ values in the range 606.03–1304.04 µg/mL and 47.81–59.25 µg/mL, respectively (Table 2), which are in accordance with previous studies of these species [18], as well as *H. perforatum* extracts [2,12,25]. It can be easily noticed that the IC_50_ values obtained for galantamine and selegiline under identical experimental conditions are significantly lower, which questions the acute effects of *Hypericum* extracts as inhibitors of AChE and MAO-B. However, in vivo studies have demonstrated the ability of *Hypericum* extracts to increase the expression of P-glycoprotein in the brain, while hyperforin is a strong inhibitor of 5-lipoxigenase [26]. Thus, the potential therapeutic effects of *Hypericum* extracts in the treatment of Alzheimer’s disease (AD) should not be neglected. Previous findings have shown significantly stronger anticholinesterase activity of hypericin (~10 folds) when compared to the flavonoids present in *Hypericum* species [27]. In our study, a moderate correlation (r = −0.41) was noticed between the abundance of hypericin and IC_50_ values describing anticholinesterase potential. However, the evaluated extracts also contained other secondary metabolites, such as chlorogenic acid and rutin, which despite having lower anticholinesterase potential than hypericin, are significantly abundant. Nevertheless, it must be stated that hypericin was previously estimated as nearly 30 times less potent as an AChE inhibitor than galantamine, which is used as a conventional drug [27]. Furthermore, previous results indicate that *Hypericum* extracts are significantly less potent as MAO-B than MAO-A inhibitors, whereas practical findings suggest that in vitro inhibition should be demonstrated in the concentration range 0.5–5 µg/mL in order to represent in vivo significance [28]. On the other hand, the IC_50_ values obtained for anti-MAO-A inhibitory activity ranged from 5.90 to 11.73 µg/mL, which is promising when compared to the inhibitory potential of moclobemide (Table 2) and corresponds to previously conducted studies. Interestingly, a statistically significant correlation (*p* < 0.05) was noticed between the anti-MAO-A potential and quantified amounts of quercetin (r = −0.89) and gallic acid (r = −0.78), which correspond to previous findings related to the bioactivity of these molecules. Namely, quercetin has demonstrated better binding affinity toward MAO-A than toward MAO-B binding sites as a consequence of the maximum π-π interaction and intramolecular H-bonds, whereas the presence of an unsaturated bond in a chromone ring is essential for MAO inhibitory activity [29,30,31]. Further, previously conducted preclinical in vivo studies have demonstrated that gallic acid inhibits MAO-A activity [29,32], while, on the other hand, hypericin and hyperforin are less probable contributors to recorded anti-MAO-A activity [33]. Generally, the obtained results represent an added value for the treatment of patients with AD since depressive episodes are the most frequent comorbidities in these patients.

##### Antihyperglycemic Potential

Regarding the increasing incidence of metabolic disorders in the world, the potential of *Hypericum* extracts to reduce postprandial glycaemia would be of high importance [34]. α-amylase and α-glucosidase are enzymes included in the initial stage of carbohydrate digestion. The potential inhibition of these enzymes would decrease the release of intestinal glucose and, consequently, decrease the glycemic load of the organism. The evaluated *Hypericum* extracts have demonstrated modest inhibitory activity of α-amylase when compared to acarbose, which was used as the positive control, but strong anti-α-glucosidase activity (Table 2). The obtained results correspond to previous studies [12,18,25,35] and indicate a strong, statistically significant correlation (*p* < 0.05) between anti-α-glucosidase activity and quantified amounts of quercetin (r = −0.80), epicatechin (r = −0.78) and phenolic acids (gallic and chlorogenic acid). Phenolic compounds have been recognized as inhibitors of the aforementioned enzymes, while in the case of α-amylase, the proposed mechanism includes the interaction of hydroxyl groups with amino acid residues at the active site (Glu233) [36]. Previous results indicate that the higher number of hydroxyl groups facilitates the inactivation of α-amylase. Furthermore, hydroxycinnamic acids contain double C-C bonds conjugated with a carbonyl group, which stabilizes the binding to the active site of α-amylase. Regarding the α-glucosidase inhibitory activity, similar to in the case of α-amylase, the higher number of hydroxyl groups increases the inhibitory effect, whereas the presence of methoxy groups in the molecule decreases it [37]. Flavonoids, especially quercetin, have been previously marked as strong inhibitors of α-amylase and α-glucosidase. Namely, hydroxyl group at C5 of the chromone ring and the double bond between C2 and C3, as well as hydroxyl group at C3′ of the phenyl substituent, are essential for anti-α-amylase activity. On the other hand, hydroxyl groups at C3 of the chromone ring and C3′ of the phenyl substituent facilitate anti-α-glucosidase activity [38]. Finally, preclinical in vivo and clinical studies indicate that *H. perforatum* extracts have the ability to reduce glycemic load. Therefore, the obtained results for evaluated *Hypericum* species that share the chemical profile with *H. perforatum* are in accordance [11].

#### 2.2.3. Chemometric Approach—Biological Potential

**Figure 2:**
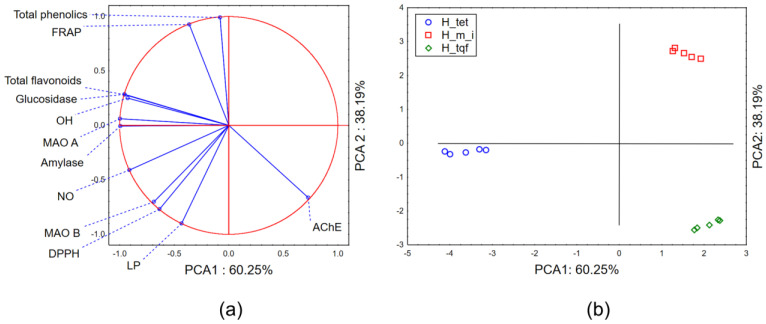
PCA—biological potential: (**a**) PCA loadings, (**b**) the position of the evaluated extracts in the space defined by the first two principal components (PCAs).

The application of PCA on the dataset combining the biological potential of the evaluated *Hypericum* extracts and the results of the preliminary chemical characterization shows that the first two principal components describe more than 98% of a sample’s variability. In terms of PCA1, most of the variability is described by the variables defining antihyperglycemic potential, anti-MAO-A potential and quantified amount of total flavonoids (Figure 2a). The shape of the variability in terms of PCA2 mostly correlates with the quantified amounts of total phenolics and the results of the antioxidant potential obtained in FRAP and the inhibition of LP assays. The position of the evaluated extracts in the space defined by the first two principal components indicates the grouping of *H. maculatum* ssp. *immaculatum* (H_m_i) and *H. triquetrifolium* (H_tqf) extracts in the positive part of the PCA1 as stronger inhibitors of the evaluated biologically important enzymes (except AChE) and stronger antioxidants than *H. tetrapterum* (H_tet) extracts. Furthermore, in the space defined by PCA2, H_m_i extracts display separative grouping as a consequence of the higher abundance of phenolic compounds and stronger antioxidant potential.

#### 2.2.4. Antibacterial and Anti-*Candida* Activity

All analyzed extracts were mostly effective against Gram-positive MRSA *S. aureus*, with the lowest activities detected at 12.5 µg/mL for all tested extracts and similarly for *Enterococcus* sp., except for *H. tetrapterum,* which showed the lowest activity (Table 3)**.** In general, MIC and MBC values were in the range 12.5–↑100 µg/mL, while among Gram-negative bacteria, *P. mirabilis* was the most susceptible, followed by *P. aeruginosa*, *E. coli* and *P. vulgaris*. Finally, the majority of the lowest MIC/MBC values were obtained at 12.5 µg/mL for both *H. triquetrifolium* and *H. maculatum* ssp. *immaculatum* extracts, both against MRSA *S. aureus* and *Enterococcus* sp., and for *H. triquetrifolium* against *P. vulgaris* and for *H. maculatum* ssp. *immaculatum* against *P. mirabilis*, which, in general, highlights the highest antibacterial effect of these two species extracts. *P. aeruginosa* isolate was multiresistent on common antibiotics, while MIC/MBC values were detected at the same concentration of 25 µg/mL for *H. maculatum* ssp. *immaculatum* and *H. triquetrifolium*. No antifungal activity was registered against *Candida* strains. The obtained results showed significantly higher antibacterial activity of the evaluated species when compared to the extract of *H. humifusum* from Tunisia (MIC values ranging from 200 to 250 μg/mL) mainly against *S. epidermidis*, *S. aureus*, and *Enterococcus faecium* [39].

Comparing the results obtained in the present study to the previous studies of the *H. lanuginosum* suggesting stronger antibacterial and antifungal activities of aqueous and methanol extracts when compared to common antibiotics, we presume that more polar extracts that contain more phenolic compounds could express better antimicrobial effects. Similar to the results for aqueous and methanolic extracts [39] that contain the highest levels of phenolics, flavonoids and tannins, we can say that in our study, the highest phenolics abundance was characteristic of *H. maculatum* ssp. *immaculatum*, followed by the highest content of caffeic, *p*-hydroxybenzoic and ferulic acids and moderate quantified amounts of rutin, hypericin and hyperforin. On the other hand, *H. tetrapterum* and *H. triquetrifolium* samples contained higher recorded amounts of hypericin, hyperforin, rutin and amentoflavone. Since the inhibitory effects of *H. perforatum* on Gram-positive bacteria have also been observed and attributed to hyperforin, the main acylphloroglucinol isolated from this plant [39], we can conclude that the highest content of this metabolite in *H. triquetrifolium* (Table 1) could explain the better antibacterial activity of this species to the others analyzed.

## 3. Materials and Methods

### 3.1. Herbal Material and Extracts Preparation

The upper aerial parts of *H. tetrapterum* and *H. maculatum* subsp. *immaculatum* were collected in 2013 in Javor Mountain (Serbia), while the *H. triquetrifolium* sample was collected in the Chalkidiki region (Greece) in the same year. The herbal material was collected at full blossom stage, and the voucher specimens (2-0400, 2-0414, 2-0664) are deposited in the BUNS Herbarium (Herbarium of the Department of Biology and Ecology, Faculty of Natural Sciences and Mathematics, University of Novi Sad). After drying, the plant material was ground and extracted by the method of maceration with 70% ethanol (m/m) for 72 h at room temperature, according to the procedure given by the EMA [40], as well as the recommendations of the European Pharmacopoeia 6th Edition [41]. The obtained extracts were filtered and evaporated to dryness in a rotary evaporator (Rotavapor R-100, Buchi). For the purpose of the determination of biological potential, 10% (m/m) solutions in water were prepared, while for the purpose of detailed chemical profiling, dry extracts were dissolved in methanol.

### 3.2. Chemical Characterization of Plant Extracts

The amount of total phenolics in the prepared extracts was determined with the Folin Ciocalteu reagent (FC reagent) spectrophotometrically using the method previously described [42]. The concentration of total phenolics was expressed in mg of gallic acid equivalents (GAE) per g of dry extract (mg GAE/g d.e.) (a standard curve for gallic acid was previously constructed). The amount of total flavonoids was also determined colorimetrically using the previously described method with aluminum chloride reagent [42], while the concentration was expressed in mg of quercetin equivalents (QE) per g of dry extract (mg QE/g d.e.) (a standard curve for quercetin was previously constructed).

For the purpose of the detailed chemical profiling of prepared extracts, two validated HPLC-DAD methods were used. The analysis was carried out on an Agilent HP 1100 instrument (Agilent, Waldbronn, Germany) equipped with a Zorbax CB-C18 column (4.6 × 150 mm, i.d., 5 µm particle). Quantification of hypericin and hyperforin was performed using the previously described method by Božin et al. [2] (Method I). Method II, for the determination of rutin, quercetin, gallic, chlorogenic, caffeic and *p*-hydroxybenzoic acids (PHB), was developed based on the report by Ziaková et al. [43]. Briefly, gradient elution was applied (3.25 min, 0% B; 8 min, 12% B, 15 min, 25% B, 15.8 min, 30% B, 25 min, 90% B, and 25.4 min, 100% B) with the flow rate of 1 mL/min, where solvent A was a 0.1% (*v*/*v*) solution of acetic acid in water and solvent B was a 0.1% (*v*/*v*) solution of acetic acid in acetonitrile. The content of secondary metabolites was expressed as µg/g of dry herbal material.

### 3.3. Antioxidant Potential

#### 3.3.1. Radical Scavenging Capacity (RSC)

The potential of the evaluated extracts to scavenge free radicals was tested in vitro against 2,2-dipheny-l-picrylhydrazil (DPPH), hydroxyl (OH) and nitroso (NO) radicals according to the previously described methods [5]. The DPPH^•^ test involved the addition of different concentrations of evaluated extracts to the DPPH^•^ solution, and the disappearance of the purple color was monitored spectrophotometrically at 515 nm. The ability of the extracts to neutralize the OH radical implied the monitoring of the degradation of 2-deoxy-D-ribose by the OH radical generated in Fenton’s reaction. As a degradation product, malonyl dialdehyde (MDA) was obtained, which was treated with thiobarbituric acid (TBA), resulting in a complex showing maximum absorption at 532 nm. The ability to neutralize NO^•^, generated from sodium nitroprusside, was measured spectrophotometrically at 546 nm after the addition of Griess’s reagent, whereby a purple coloration was formed.

The degree of neutralization of the tested free radicals expressed in percentages was calculated according to Equation (1):

RSC (%) = 100 × (A_blank_ − A_sample_/A_blank_) (1)

#### 3.3.2. Inhibition of Lipid Peroxidation (LP)

The capacity of the evaluated extracts to inhibit the process of lipid peroxidation was investigated based on research published by Kladar et al. [5]. Liposome emulsion was used as a model of cell membranes, and the OH radicals that cause this process were generated by Fenton’s reaction. As a product of the degradation reaction, MDA was obtained, which with thiobarbituric acid forms a complex showing maximum absorption at 532 nm.

The percentage of LP inhibition was calculated by Equation (2):

I (%) = (A_o_ − A_1_)/A_o_ × 100 (2)

where A_o_ was the absorbance of the control reaction (reaction mixture without extract), and A_1_ was the absorbance of the examined samples

The results of the antioxidant potential evaluation were compared with positive controls—recognized antioxidants, such as ascorbic acid (AA), butylated hydroxytoluene (BHT), propyl gallate (PG) and quercetin dihydrate (QDH), evaluated under the same experimental conditions.

#### 3.3.3. Ferric Reduction Antioxidant Potential

Based on the method previously described by Lesjak et al. [44], the ability of extracts to reduce Fe^3+^ to Fe^2+^ was investigated. Reduced Fe^2+^ reacts with 2,4,6-tripyridyl-S-triazine (TPTZ) and forms a blue-colored complex with an absorption maximum at 593 nm. The results were expressed as mg of ascorbic acid equivalents per g of dry extract weight (mg AAE/g d.e.) based on the previously assessed antioxidant potential of ascorbic acid under the same experimental conditions.

### 3.4. Inhibition of Biologically Important Enzymes

#### 3.4.1. Inhibition of Acetylcholinesterase

The ability of the extracts to inhibit acetylcholinesterase was determined spectrophotometrically using the modified Ellman’s method [2]. Sodium phosphate buffer (pH = 7.2), color indicator (5,5′-dithiobis-(2-nitrobenzoic acid)–DTNB, containing NaHCO_3_), plant extracts and acetylcholinesterase solution were mixed in the test tube and left at room temperature for 15 min. The final activity of the enzyme in the reaction mixture was 8.15 U/L. After that, the substrate, acetylthiocholine iodide, was added, and the change in the absorbance at 405 nm was monitored for 3 min. The percentage of enzyme activity inhibition was calculated based on a control mixture containing distilled water instead of the extract, in which the enzyme was considered to have reached 100% activity. Galantamine was used as a positive control.

#### 3.4.2. Inhibition of Monoamine Oxidase A (MAO-A) and Monoamine Oxidase B (MAO-B)

The potential of the extracts to inhibit human recombinant MAO-A and MAO-B was determined spectrofluorimetrically according to the study performed by Samoylenko et al. [45]. During the test, the reaction mixture contained the appropriate enzyme (MAO-A or MAO-B), phosphate buffer, kynuramine, as well as increasing concentrations of extracts. Kynurenine, which was used as a substrate for the aforementioned enzymes, after enzymatic degradation turns into 4-hydroxyquinoline. During MAO-A inhibition testing, the final enzyme and substrate concentrations in the reaction mixture were 5 μg/mL and 80 μM, respectively, while in the case of MAO-B inhibition, the enzyme and substrate concentrations were 10 μg/mL and 50 μM. For the control test, instead of the extract, the buffer was added to the reaction mixture. Moclobemide and selegiline were used as positive controls for MAO-A and MAO-B inhibition.

#### 3.4.3. Inhibition of α-Amylase

The ability of the extracts to inhibit α-amylase was studied according to the spectrophotometric method described by Kladar et al. [5]. For the purposes of the experiment, porcine 𝛼-Amylase (final reaction mixture activity 0.6 U/mL), Starch azure^®^ (Sigma Aldrich, Wien, Austria) and sodium phosphate buffer (pH = 7.2) with NaCl were used. Two different concentrations of extracts were added to the reaction mixture; after incubation for 10 min, the reaction was stopped by the addition of acetic acid (50%, m/m). The inhibition percentage was calculated based on the control measurement that contained water instead of the extract. Acarbose was used as a positive control.

#### 3.4.4. Inhibition of α-Glucosidase

The ability of the tested extracts to inhibit the activity of α-glucosidase isolated from *Saccharomyces cerevisiae* was determined by the official Sigma Aldrich method [46]. The reaction mixtures contained potassium phosphate buffer (pH = 6.8), glutathione solution (reduced form), enzyme α-*glucosidase* (final activity in the reaction mixture was 7.6 U/L) and *p*-nitrophenyl-α-D-glucoside (PNP-Gluc) as substrate. After the addition of two different concentrations of the tested extracts and incubation at 37 °C for 20 min, the reaction was stopped with Na_2_CO_3_ solution. The percentage of inhibition was calculated based on the control solution, which contained distilled water instead of the tested extracts, and was considered to exhibit 100% enzyme activity. Acarbose was used as a positive control.

#### 3.4.5. Calculations of Enzymes Inhibitory Activity

The percentage of evaluated enzyme inhibition was calculated according to Equation (3):

I (%) = 100 − (A_sample_/A_control_) × 100 (3)

where A_sample_ was the absorbance of the reaction mixture containing extract, and A_control_ was the absorbance of the control tube containing no extract considered for 100% of enzyme activity.

### 3.5. Antimicrobial Activity

The antibacterial activity was evaluated by the double micro-dilution method for the determination of minimum inhibitory (MIC) and bactericidal concentration (MBC) against six clinical bacterial strains and two *Candida* strains isolated from pregnant women with symptoms of vaginal infections according to the standard CLSI procedure and previously described methods [47,48]. Two Gram-positive (*S. aureus* MRSA, *Enterococcus* sp.) and four Gram-negative strains (*Escherichia coli*, *Proteus mirabilis*, *Proteus vulgaris* and *Pseudomonas aeruginosa)* were used to determine the antibacterial activity. The testing of antifungal activities was determined on *C. albicans* strains. Two laboratory strains were obtained from the Department of Biology and Ecology, Faculty of Sciences, University of Novi Sad, and four clinical isolates were obtained from the Faculty of Medicine, Clinical Center of Vojvodina, Department of Obstetrics and Gynecology, isolated during regular gynecological examination in women. Their use was approved by the Ethics Committee of the Faculty of Medicine. Microtiter plates were incubated in a thermostat for 24 h at 37 °C, and MIC and MBC were determined. The disk-diffusion method was applied for bacterial susceptibility of standard antibiotics (Himedia, Einhausen, Germany): erythromycin, clindamycin, ciprofloxacin, gentamicin, ofloxacin, chloramphenicol and levofloxacin.

### 3.6. Data Processing

The obtained data were processed using Microsoft Office Excel (v2019) and Tibco Statistica (v13.5). The results were analyzed by means of descriptive statistics, as well as by application of univariate and multivariate statistical analysis—principal component analysis (PCA). The correlations between the obtained results were assessed by application of the Pearson correlation coefficient, while the statistical level of significance was kept at *p* = 0.05. The differences between the evaluated species were analyzed by the application of Kruskal–Wallis ANOVA followed by multiple comparisons of mean ranks, whereas the differences were considered statistically significant if *p* < 0.05. Principal component analysis is a dimension reduction technique that enables a better understanding of dataset patterns of variability in the space described by a lower number of dimensions, principal components, which correlate to the original variables used to describe dataset variability.

## 4. Conclusions

The conducted study has indicated a strong qualitative and quantitative resemblance of the chemical profiles of the examined *Hypericum* species with the official biological source of *Hyperici herba* (*H. perforatum*). Consequently, the studied biological activities showed similar potential to *H. perforatum*. Specifically, the evaluated species were good scavengers of free radicals, as well as strong inhibitors of MAO-A and α-glucosidase, which demonstrates their in vitro potential for the treatment of depression and diabetes mellitus type 2. All analyzed *Hypericum* species were of great potential for the treatment of infections caused by Gram-positive pathogens, while of particular importance was the anti-staphylococcal effect of *H. maculatum* ssp. *immaculatum*. Furthermore, the high anti-pseudomonas activity of *H. triquetrifolium* requires particular attention regarding the general antimicrobial resistance of this strain. The aforementioned aspects highlight the importance of further conducting in vivo preclinical and clinical studies on the evaluated *Hypericum* species in order to elucidate the clinical significance and safety of their application.

## Figures and Tables

**Table 1:** Chemical characterization of the evaluated species.

Sample		*H. tetrapterum*	*H. maculatum* ssp. *immaculatum*	*H. triquetrifolium*
Variable				
Total phenolics (mg GAE)/g d.e.		137.77 ± 10.1 ^a^	194.24 ± 14.12 ^a^	83.52 ± 7.60 ^a^
Total flavonoids (mg QE/g d.e.)		58.17 ± 4.51 ^b^	37.01 ± 3.16 ^b^	24.76 ± 2.36 ^b^
Dry extract yield (%)		12.77 ± 1.11 ^c,d^	19.30 ±1.87 ^c^	19.89 ± 1.98 ^d^
Class of compounds	Compound	µg/g dry herb		
Naphthodianthrones	Hypericin	450.51 ± 32.11 ^e^	52.71 ± 4.23 ^e^	185.16 ± 14.36 ^e^
Phloroglucinols	Hyperforin	1235.02 ± 56.78 ^f^	278.9 ± 25.64 ^f^	1563.1 ± 114.65 ^f^
Biflavonoids	Amentoflavone	135.06 ± 11.12 ^g^	n.d. ^g^	72.26 ± 5.47 ^g^
Flavonoids and flavonoid glycosides	Apigenin	n.d. ^h^	0.82 ± 0.11 ^h,i^	n.d. ^i^
	Naringenin	n.d. ^j^	n.d. ^k^	249.83 ± 19.21 ^j,k^
	Rutin	550.93 ± 36.78 ^l,m^	222.5 ± 23.56 ^l^	278.07 ± 22.11 ^m^
	Quercetin	150.47 ± 9.45 ^n^	183.09 ± 14.32 ^n^	173.88 ± 13.28
	Epicatechin	n.d. ^o^	n.d. ^p^	390.09 ± 32.06 ^o,p^
Phenolic acids	Ferulic acid	n.d. ^q^	259.08 ± 23.56 ^q,r^	n.d. ^r^
	Gallic acid	62.04 ± 4.15 ^r^	66.52 ± 6.14 ^r^	77.07 ± 7.16
	Chlorogenic acid	n.d. ^s,t^	127.19 ± 16.78 ^s^	105.35 ± 9.25 ^t^
	Caffeic acid	39.74 ± 3.65 ^u^	125.12 ± 13.54 ^u,w^	45.47 ± 4.14 ^w^
	p-hydroxybenzoic acid	46.71 ± 3.78 ^x^	219.47 ± 22.65 ^x,y^	56.75 ± 5.14 ^y^

The results are expressed as an average value ± standard deviation (Xm ± S.D.) of three repeated measurements. The identical lower-case letters denote statistically significant differences (*p* < 0.05) between evaluated species; n.d.—not detected.

**Table 2:** Biological potential of investigated *Hypericum* species.

Sample	*H. tetrapterum*	*H. maculatum* ssp. *immaculatum*	*H. triquetrifolium*	Positive Control
Variable	RSC_50_ (µg/mL)			
DPPH	3.54 ± 0.33 ^a^	1.93 ± 0.13 ^a^	3.14 ± 0.29 ^a^	QDH, RSC_50_ = 1.01 ± 0.08 PG, RSC_50_ = 0.65 ± 0.05
NO	32.17 ± 3.11 ^b^	12.11 ± 1.98 ^b^	19.24 ± 1.57 ^b^	PG, RSC_50_ = 8.87 ± 0.79
OH	58.74 ± 4.26 ^c^	55.00 ± 4.87 ^c^	51.74 ± 4.23 ^c^	BHT, IC_50_ = 0.03 ± 0.00 AA, IC_50_ = 2.21 ± 0.17 PG, IC_50_ = 10.11 ± 0.69
LP	n.d. ^d^	514.96 ± 36.75 ^d,e^	n.d ^e^	BHT, IC_50_ = 7.99 ± 0.69
FRAP (mg AAE/g d. e.)	162.18 ± 12.98 ^f^	176.75 ± 14.25 ^f^	113.76 ± 10.58 ^f^	/
AChE	606.03 ± 54.23 ^g^	774.89 ± 56.92 ^g^	1304.04 ± 116.88 ^g^	Galantamine IC_50_ = 9.11 ± 0.64
MAO-A	11.73± 0.88 ^h^	5.90± 0.26 ^h^	4.79 ± 0.32 ^h^	Moclobemide IC_50_ = 0.71 ± 0.08
MAO-B	59.25 ± 5.23 ^i^	47.81 ± 4.23 ^i^	55.15 ± 4.13 ^i^	Selegiline IC_50_ = 0.22 ± 0.02
α-amylase	8440.34 ± 654.28 ^j^	1270.62 ± 115.32 ^j^	616.04 ± 53.87 ^j^	Acarbose IC_50_ = 5.35 ± 0.72
α-glucosidase	22.43 ± 2.11 ^k^	14.56 ± 1.12 ^k^	9.94 ± 0.65 ^k^	Acarbose IC_50_ = 48.76 ± 3.45

Antioxidant potential (neutralization potential of DPPH, NO and OH radicals, inhibition of lipid peroxidation (LP) process, ferric reduction antioxidant potential (FRAP)) and inhibition of biologically important enzymes—acetylcholinesterase (AChE), monoamine oxidases A and B (MAO-A and MAO-B), α-amylase and α-glucosidase). The results are expressed as an average value ± standard deviation (Xm ± S.D.) of three repeated measurements. The identical lower-case letters denote statistically significant differences (*p* < 0.05) between evaluated species; QDH—quercetin dihydrate, PG—propyl gallate, BHT butylated hydroxytoluene, AA—ascorbic acid, n.d.—not detected.

**Table 3:** Antimicrobial activity of extracts of *Hypericum* species (µg/mL) and common Antibiotics (mm).

Agent	*H. tetrapterum*		*H. maculatum* ssp. *immaculatum*		*H. triquetrifolium*		Antibiotics (mm)							
Microbe	MIC	MBC	MIC	MBC	MIC	MBC	E (15 µg)	LEV (5 µg)	DA (2 µg)	KF (20 µg)	CIP (5 µg)	CN (10 µg)	OFX (5 µg)	C (30 µg)
*S. aureus* ^H^ *MRSA*	12.5	25	12.5	12.5	12.5	12.5	22.5	27.5	25	35	24.5	19	26	25
*E. coli* ^L^	100	100	25	25	50	100	/	35	/	16	25.5	15	22.5	23.5
*P. mirabilis* ^H^	12.5	50	12.5	12.5	50	50	/	20	/	/	20	/	17	10
*P. aeruginosa* ^H^	25	50	25	25	25	25	/	/	/	/	/	/	/	/
*Enterococcus* sp. ^L^	100	100	12.5	25	12.5	25	12.5	24	/	20	20.5	10.5	19.5	25.5
*P. vulgaris* ^L^	↑100	↑100	50	50	12.5	12.5	/	29.5	21	/	31	16.5	22.5	20
*Candida* ^L^	/	/	/	/	/	/	/	/	/	/	/	/	/	/
*Candida* ^H^	/	/	/	/	/	/	/	/	/	/	/	/	/	/

Legend: E—erythromycin, LEV—levofloxacin, DA—clindamycin, KF—ceftiofur, CIP—ciprofloxacin, CN—gentamicin, OFX—ofloxacin, C—chloramphenicol, ^H^—human isolate, ^L^—laboratory strain.

## Data Availability

Not applicable.
